# Experimental Investigations on the Effects of Carbon and Nitrogen Sources on Concomitant Amylase and Polygalacturonase Production by *Trichoderma viride* BITRS-1001 in Submerged Fermentation

**DOI:** 10.1155/2012/904763

**Published:** 2012-07-15

**Authors:** Arotupin Daniel Juwon, Ogunmolu Funso Emmanuel

**Affiliations:** ^1^Department of Microbiology, Federal University of Technology Akure, PMB 704, Akure, Ondo State, Nigeria; ^2^Department of Microbiology and Biotechnology, Afe Babalola University, Ado-Ekiti, Ekiti State, Nigeria

## Abstract

The paper investigates the effects of different commercial carbon and nitrogen sources on the concomitant synthesis of amylase and polygalacturonase enzymes with the aim of optimizing them for maximal enzyme production. The microorganism used in this work was the fungus *Trichoderma viride* BITRS-1001, which had been previously identified as a highly active producer of amylase and polygalacturonase enzymes. The results showed that the different commercial carbon and nitrogen substrate significantly affected the concomitant syntheses of amylase and polygalacturonase in culture media supplemented with the different commercial carbon and nitrogen substrates. The result obtained suggested that for optimal and concomitant synthesis of the enzymes by *Trichoderma viride* BITRS-1001 in submerged fermentation, minimal medium supplemented with maltose and casein were the carbon and nitrogen substrates of choice.

## 1. Introduction

Microbes are rich sources of enzymes [1]. In nature, they have been endowed with vast potentials to produce array of enzymes, which have been exploited commercially over the years. Traditionally, enzymes have been extracted from plants and animals. However, microbial enzymes have formed the basis of commercial enzyme production. In recent years, the potential of using microorganisms as biotechnological sources of industrially relevant enzymes has stimulated interest in the exploration of extracellular enzymatic activity in several microorganisms isolated from different environments owing to several reasons [2–7].

Amylase (EC 3.2.1.1) and polygalacturonase (EC 3.2.1.15) from microbial origin have high biotechnological interest such as in the processing of foods, manufacturing of detergents, textiles, pharmaceutical products, medical therapy, in molecular biology, and in many industrial processes as reviewed in [4, 6, 8–22]. While amylase has been reported to have approximately 25% of the enzyme market of industrial enzymes [17, 23, 24], microbial pectinases have been reported to account for 25% of the global food enzymes sales [4].

The synthesis of these enzymes by microorganisms has been reported to be highly influenced by factors such as carbon sources, temperature, pH, and operating parameter such as incubation time in submerged culture [25, 26]. Factors like carbon, nitrogen sources and their concentrations have always been of great interest to researchers in the industry for the low-cost media design. It is also known that 30–40% of the production cost of industrial enzymes is estimated to be the cost of growth medium. Therefore, it is of great significance to optimize the conditions for cost-efficient enzyme production [26].

However, investigations on the impact of carbon and nitrogen supplements revealed that not all carbon and nitrogen sources would act as enhancer for simultaneous production of these enzymes in a single fermentation system. Unlike in single-enzyme production, the roles of supplements become very critical in multienzyme production as not many supplements enhance simultaneous production of all enzymes in a single bioreactor [27]. Production of amylase and polygalacturonase in single fermentation can be particularly effective and useful for industries where both these enzymes are used together, such as food, animal feed, and textile. The present study was therefore aimed at the experimental investigations on the effect of carbon and nitrogen sources of the concomitant synthesis of amylase and polygalacturonase enzymes by *Trichoderma viride* BITRS-1001 in a single fermentation.

## 2. Materials and Methods

### 2.1. Source of Microorganism

The fungus strain used in this work was *Trichoderma viride* BITRS-1001, which had been previously identified as a highly active producer of amylase and polygalacturonase enzymes in the research laboratory of the Department of Microbiology, The Federal University of Technology Akure, Nigeria [28]. The culture was maintained on Sabouraud Dextrose Agar slants incorporated with 0.1% tetracycline kept at 4°C and subcultured at regular intervals.

#### 2.1.1. Cultural Conditions and Concomitant Production of Amylase and Polygalacturonase in Submerged Cultivation (SmC)

The concomitant production of the hydrolytic enzymes (amylase and polygalacturonase) was carried out in 250 mL conical flasks each containing 50 mL modified basal medium of Arotupin [29]. The composition of the basal medium included peptone 1 g; KH_2_PO_4_ 1.05 g; NaNO_3_ 4 g; MgSO_4_ 7H_2_O 0.1 g; Na_2_HPO_4_ 2 g; sucrose 20 g and distilled water 1000 mL. The medium was adjusted to a pH of 6.00. A sterile cork borer of 15 mm diameter was used to cut a disc from the advancing edge of a 5 days old fungal isolate. The disc was used to inoculate the medium. Fermentation carried out at 30°C for the fungal isolates in a Gallenkamp BKS-350-0010 orbital incubator shaker operating at 80 rpm for 10 days. The following parameters were monitored daily: growth (usually estimated as the dry weight of mycelium per 50 mL), pH, amylase, and polygalacturonase (PG) activities.

To investigate the influence of carbon and nitrogen sources on the enzyme activities of *T. viride* BITRS-1001 in submerged cultivation, sucrose was replaced with fructose, maltose, lactose, and starch, while the mixture of sodium nitrate and peptone was substituted with peptone, casein, sodium nitrate, and urea.

#### 2.1.2. Growth Determination of Fungal Isolate

The method of Narasimha et al. [30] was employed. The mycelia growth produced in the liquid culture medium was determined by dry weight measurement. Whatman number 1 filter paper was dried to constant weight at 80°C, and the weight noted. The content of the flask was filtered through the filter paper to separate the mycelia mat and the culture filtrate. The biomass of the culture (residue) was dried until a constant weight was obtained. The growth yield per 50 mL of broth was determined using a Mettler balance (PM 400). The growth was calculated, thus

(1) Growth  (mg/50 mL)=Weight  of  culture+filter  paper−initial  weight  of  filter  paper.

#### 2.1.3. Determination of the pH of the Culture Filtrate

The pH value of the culture filtrates was obtained by using an electronic pH meter, Hanna pH209 that was initially standardized with appropriate buffer solutions of pH 4, 7, and 9. The electrode of the standardized pH meter was inserted into the crude filtrate of the isolate. The values were immediately read on the meter record and values recorded. This was done throughout the period of the experimental setup [31].

#### 2.1.4. Assay for Amylase Activity of the Culture Filtrate

The amylase activity of the culture filtrate was determined as described by Sudharhsan et al. [24]. Crude culture filtrate was used as enzyme sample. A 0.5 mL of culture filtrate was boiled in a water bath (100°C) for 20 minutes in order to inactivate the enzyme and then cooled suddenly under tap. Both heat treated and active samples were taken for the assay. 1% starch substrate was prepared freshly in 0.1 M phosphate buffer (pH 6.0). The reaction mixture containing 500 *μ*L of substrate (starch) and 500 *μ*L of enzyme solution was incubated at 37°C for 15 minutes for enzymatic reaction. After incubation, 1 mL of DNSA was added and heated for 15 minutes in a boiling water to obtain a coloured reacted mixture. Absorbency of the solution was measured at 550 nm using UV-VIS spectrophotometer (UNICO 1100RS spectrophotometer).

The heated enzyme mixture served as a blank. One unit of amylase enzyme activity was defined as the amount of starch hydrolyzed during 15 minutes incubation at 37°C for 1 mL of extract. Serial dilutions of glucose were treated in the same manner and the absorbance reading was taken and used to plot a standard curve for glucose. The unknown amount of reducing sugar in each test sample was extrapolated from the standard curve [32].

### 2.2. Assay for Polygalacturonase Activity of the Culture Filtrate

Polygalacturonase (PG) activity of the culture filtrate was assayed by measuring the amount of reducing sugar released in the reaction mixture. The reaction mixture consisted of 1 mL of 1.2% (w/v) pectin in 1 mL of 0.1 M citrate-phosphate buffer of pH 5.0 and 1 mL of crude filtrate (crude enzyme solution). Control experiment tubes contained the same amount of substrate and 1 mL of the crude filtrate (crude enzyme solution) boiled for 15–20 minutes. Both the experimental and control tubes were incubated at 35°C for 3 hours. The reducing sugar released into the reaction mixture was determined by the method of 3,5-Dintrosalicyclic acid (DNSA) reagent [31]. One unit of polygalacturonase activity was defined as the amount of enzyme in 1 mL that would liberate reducing sugar equivalent to 1 *μ*g galacturonic acid per minute under the specific conditions of reaction.

A 3 mL of DNSA reagent (NaOH 10 g; Na/K-tartrate 20 g; 3,5-dinitrosalicyclic acid 10 g and distilled water 1000 mL) was added to 1 mL of each of the test sample in the test tubes. The mixture was properly mixed and heated in boiling water for 15 minutes and cooled in tap water. The absorbance was taken at 540 nm with a UNICO 1100RS spectrophotometer. Serial dilutions of galacturonic acid were treated in the same manner and the absorbance reading taken and used to plot a standard curve for polygalacturonase (PG). The unknown amount of reducing sugar in each test sample was extrapolated from the standard curve.

### 2.3. Statistics

The numerical data obtained during the investigations were subjected to analysis of variance and inferences were made at 95% confidence limits using the SPSS 15.0 software package. Duncan's new multiple range test was used to separate means.

## 3. Results

### 3.1. Effects of Different Carbon Sources on Growth and Enzyme Activities of *T. viride* BITRS-1001

**Figure 1:**
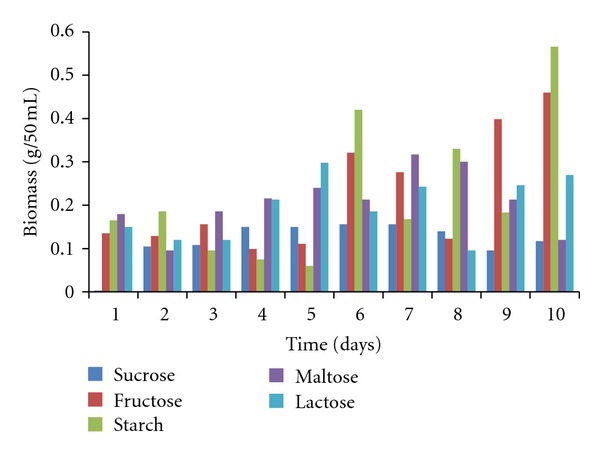
Effect of different carbon sources on the biomass of *T. viride* BITRS-1001 in submerged culture (SmC).

**Figure 2:**
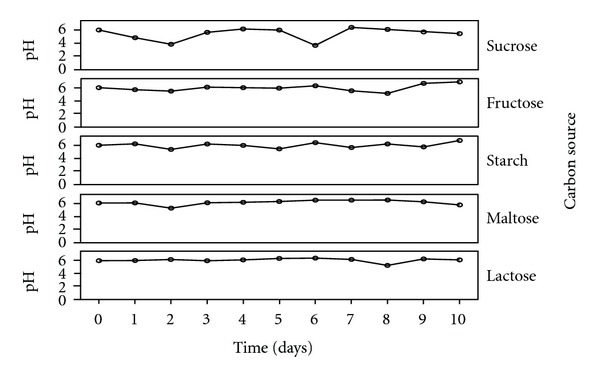
pH variations in the culture media during fermentation in submerged culture (SmC).

**Figure 3:**
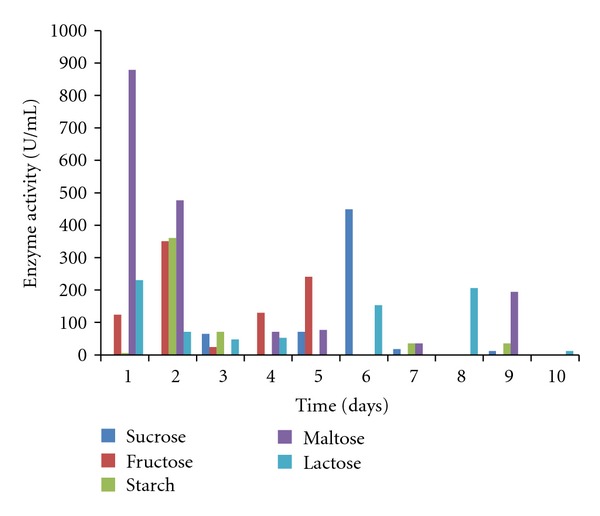
Effect of different carbon sources on amylase activity of *T. viride* BITRS-1001 in submerged culture (SmC).

**Figure 4:**
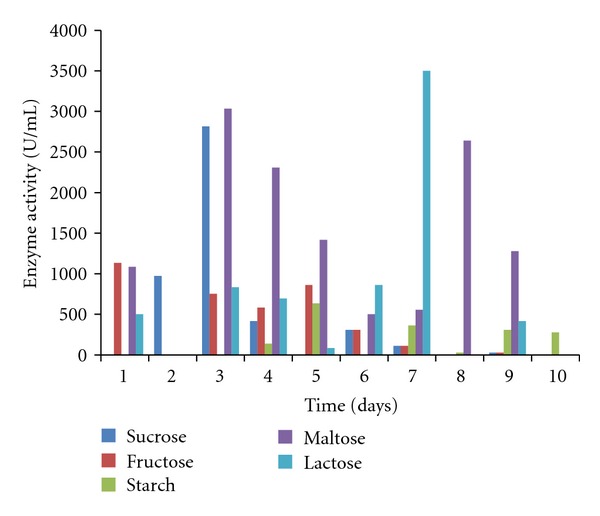
Effect of different carbon sources on polygalacturonase activity of *T. viride* BITRS-1001 in submerged culture (SmC).

The mycelia dry weight, pH values, protein content, amylase, and polygalacturonase activities of the culture filtrates of *T. viride *30°C ± 2 were determined using various commercial carbon sources namely starch, lactose, fructose, maltose, and sucrose. All the commercial carbon sources supported good growth of the fungal isolates as well as the concomitant production of the enzymes of interest. *T. viride* had the highest biomass yield of 0.566 g/50 mL of culture in starch, followed by 0.460 g/50 mL in fructose, 0.317 g/50 mL in maltose, and 0.298 g/50 mL in lactose, while sucrose had the least biomass yield of 0.1560 g/50 mL culture medium (Figure 1). The pH values of the culture filtrate ranged from 3.60 to 6.39 for sucrose, 5.12 to 6.87 for fructose, 5.35 to 6.73 for starch, 5.20 to 6.45 for maltose, and 5.24 to 6.40 for lactose (Figure 2). The highest amylase activity of *T. viride* was recorded within 24 hours from maltose medium with activity of 878.33 U/mL, sucrose (448.667 U/mL on the 6th day), starch (360.16 U/mL on the 2nd day), and fructose (350.5 U/mL on the 2nd day) while lactose has an activity of 230 U/mL within 24 hours (Figure 3). In the case of polygalacturonase, lactose medium had polygalacturonase activity of 3500 U/mL on the 7th day, maltose (3033 U/mL on the 3rd day), fructose (1133.33 U/mL on the 1st day), starch (633.33 U/mL on the 5th day), and sucrose with activity 2816.7 on the 3rd day (Figure 4).

### 3.2. Effects of Different Nitrogen Sources on Growth and Enzyme Activities of *T. viride *BITRS-1001

**Figure 5:**
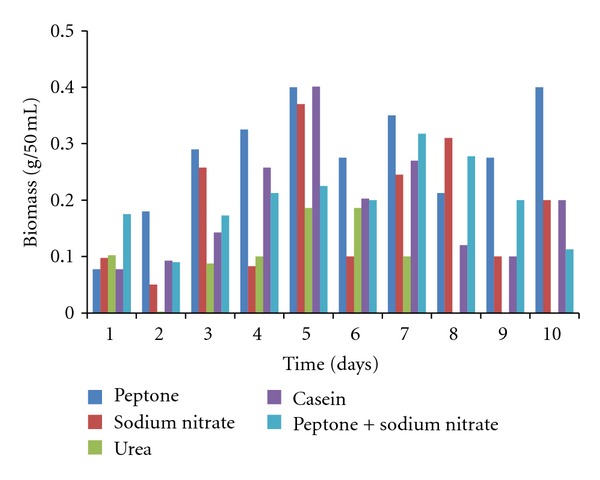
Effect of different nitrogen sources on the biomass of *T. viride* BITRS-1001 in submerged culture (SmC).

**Figure 6:**
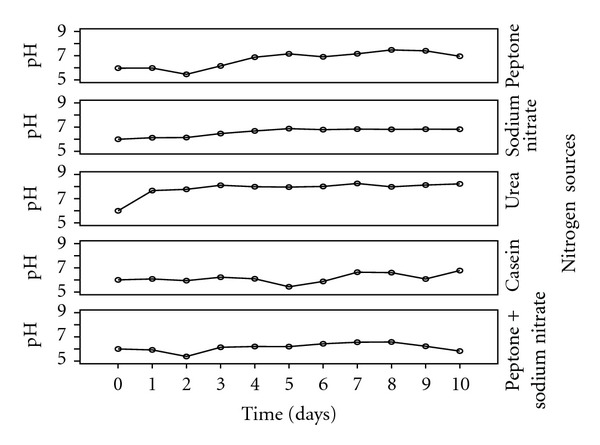
pH variations in the culture media during fermentation of *T. viride* BITRS-1001 in submerged culture (SmC).

**Figure 7:**
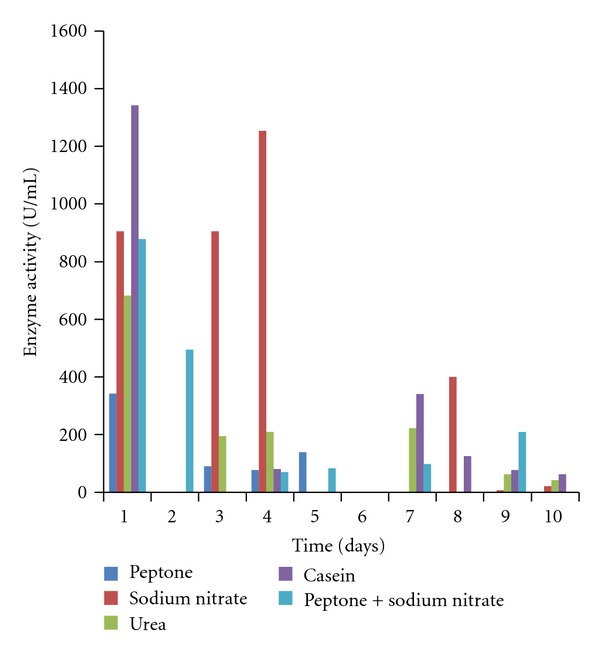
Effect of different nitrogen sources on amylase activity of *T. viride* BITRS-1001 in submerged culture (SmC).

**Figure 8:**
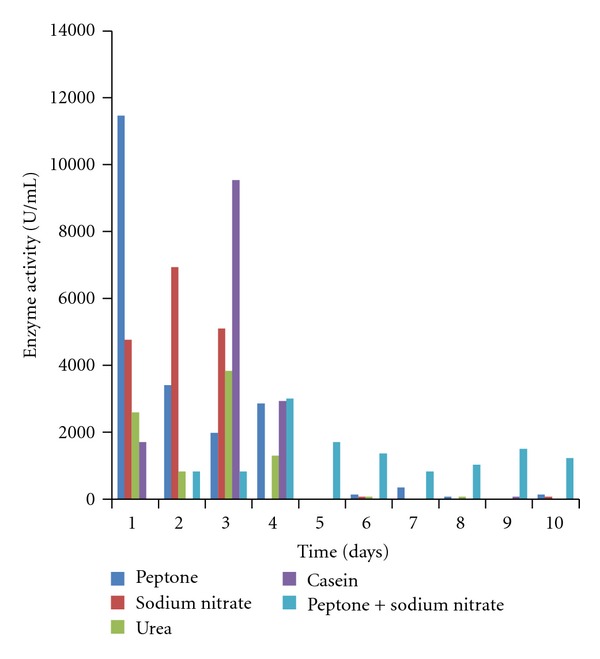
Effect of different nitrogen sources on polygalacturonase activity of *T. viride* BITRS-1001 in submerged culture (SmC).

**Figure 9:**
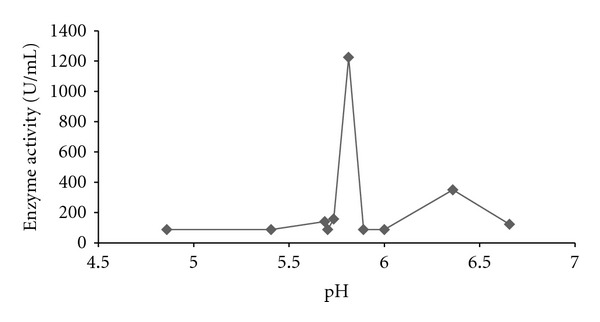
Relationship between changes in the pH of the culture media with amylase activity of *T. viride* BITRS-1001 in casein supplemented medium.

**Figure 10:**
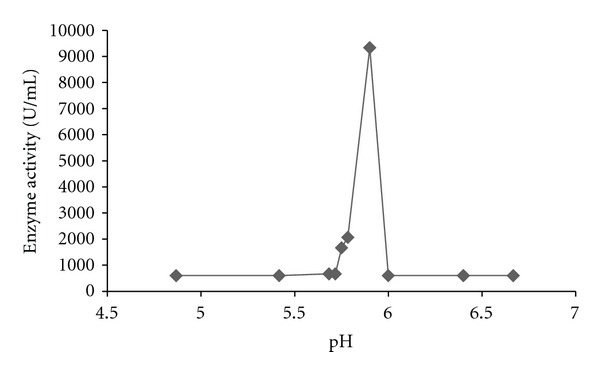
Relationship between changes in the pH of the culture media with polygalacturonase activity of *T. viride* BITRS-1001 in casein supplemented medium.

The effects of different nitrogen sources on the growth and enzyme production are shown in Figures 5–8. The various nitrogen sources stimulated the growth of the fungus and the production of amylase as well as polygalacturonase in varying degrees. *T. viride* BITRS-1001 grew best in media containing casein and peptone as nitrogen sources with mycelia growth of 0.401 g/50 mL on day 5 and 0.400 g/50 mL on day 10, respectively. Peptone + sodium nitrate had the maximum biomass growth of 0.317 g/50 mL on day 7, sodium nitrate (0.310 g/50 mL on day 8), and urea with a value of 0.186 g/50 mL on day 6 of the culture medium (Figure 5). The pH variations in the culture media during fermentation in submerged culture (SmC) are indicated in Figure 6. The pH values of the culture medium during the period of incubation ranged from 5.45 to 7.47 for peptone, 6.00 to 6.88 for sodium nitrate, 6.0 to 8.26 for urea, 5.43 to 6.77 for casein, and 5.38 to 6.58 for peptone + sodium nitrate. The relationships between the varying pH and the measured amylase and polygalacturonase activities in the crude filtrate are also shown in Figures 9 and 10. The highest amylase activities per nitrogen sources are as follows. Casein medium 1341.667 U/mL, peptone + sodium nitrate 878.33 U/mL, urea 682 U/mL, and peptone 342.0 U/mL, respectively, within 24 hours, while sodium nitrate had amylase activity of 1253.33 U/mL on the 4th day (Figure 7). For the polygalacturonase activities of *T. viride* BITRS-1001 in the various nitrogen substrates, peptone had the highest polygalacturonase activity of 11466.67 U/mL within 24 hours, sodium nitrate (6933.3 U/mL on the 2nd day), casein (9533.33 U/mL on the 3rd day), peptone + sodium nitrate (3000 U/mL on the 4th day), and urea 3833.33 U/mL on the 3rd day (Figure 8).

## 4. Discussion

The result of this investigation showed that the fungus *T. viride* BITRS-1001 had the ability to utilize the various carbon and nitrogen sources as good substrates for growth as well as for the concomitant production of amylase and polygalacturonase in submerged cultivation. Fungi being heterotrophs obtain their required nutrients from the organic matter in the environment through the presence of efficient and extensive systems of powerful enzymes. Thus, they are able to utilize complex carbon sources as their energy source [33, 34]. Of the carbon sources tested, starch (a polysaccharide) supported the maximum biomass yield followed by fructose (monosaccharide). The least biomass yield was observed in the disaccharides in the following order: maltose, lactose, and sucrose. The dominance of polysaccharides over disaccharides and monosaccharides in supporting the growth of fungi had earlier being reported by [35] and Akinyosoye et al. [2], who reported that starch supported the maximum biomass yield of *Geotrichum candidum* and *Phoma sorghina* better than disaccharides (maltose and lactose), monosaccharides (glucose, fructose, and galactose). Arotupin [36] on the contrary however reported that starch supported the least biomass yield of *Aspergillus* spp. grown in submerged cultivation. The observed maximum biomass yield of *T. viride* BITRS-1001 on starch supplemented medium may possibly be due to the fact that starch is the most abundant organic carbon source in the environment serving as the major reserve carbohydrate of all higher plants, with the fact that it is extensively degraded by *α*-amylase, which is readily produced by the fungus [8].

In the course of the investigation, it was observed that the pH of the culture media varied over a wide range of values within the acidic region on the pH scale. Fungi generally alter the pH of the medium in which they grow, due to uptake of the anions or cations in the medium [34, 37]. Therefore, the varied changes witnessed in the pH values of the culture media may be as a result of the utilization of some compounds in the culture media. Nonetheless, the confinement of the variations in pH within the acidic region on the pH scale is in consonance with previous reports that fungi are generally acidophilic [34]. In relating the changes in the pH of the culture media with the production of the enzymes in question using scatter plots, it was observed that the alterations in the pH of the culture media produced significant effects on the activities of the different enzymes investigated. The activity of certain fungi extracellular digestive enzymes had earlier been reported to be affected by the pH of their culture media [38]. Although *T. viride* BITRS-1001 grew over a wide range of pH, the highest enzyme activities were noticed at pH values 5.78 for amylase and 5.99 for polygalacturonase. pH values below or above the observed range resulted in decrease in the activities of the two enzymes.

Earlier investigations reported optimum amylase production by *Aspergillus ochraceus* at pH 5.5 [39], *Streptomyces albidoflavus *at pH 6.5 [40], and *Aspergillus awamori* at pH 5.5 [27]. Optimum polygalacturonase activity by *A. niger* occurred at pH 5.5 [26]. Changes in pH do affect the affinity of enzymes for substrates, especially when the active site has been altered. A decreased saturation of the enzyme with the substrate as a result of the decrease in affinity may be responsible for the decline in either side of the optimum or may be due to the effect of pH on the stability of enzymes. This leads to a considerable denaturation and subsequent inactivation of the enzymes [29]. The reduction observed in the enzyme activity of *T. viride* BITRS-1001 at pH values other than the optimal pH could also be attributed to a probable change in the state of the ionic groups involved in the maintenance of the active conformation of the enzymes. Extreme pH has been reported to initiate chemical reactions that can alter, cross-link, or destroy amino residues of the protein molecules resulting in irreversible inactivation. Since enzymes are proteins, variations in pH will ultimately affect the ionic characters of the important acidic and basic groups in the active center which are essential for the catalytic activities of the enzymes [29].

In addition, the results from this investigation on the effects of the different commercial carbon substrate tested revealed varied responses of *T. viride* BITRS-1001 in concomitantly producing amylase and polygalacturonase in culture media supplemented with the different commercial carbon substrates. Maximum amylase and polygalacturonase activities were recorded on maltose supplement medium within 24 hours and 72 hours, respectively. The addition of carbon sources in the form of either mono saccharides or polysaccharides had earlier been reported to influence the production of enzymes *in vitro* [24]. Glucose was reported to have supported amylase activity in *Aspergillus* sp. JG1 12 [6], glucose and lactose in *A. awamori* [27], and starch in *Aspergillus* spp. [41], while pectin has been reported to induce the polygalacturonase activity in submerged culture [9, 42]. However, the ability of maltose to support maximum activities of amylase and polygalacturonase within the shortest incubation time is desirable in comparison to the other sugars tested in industrial processes. Thus, maltose was chosen as the carbon substrate of choice for the remainder of the investigation in testing for the effect of different nitrogen substrates on the concomitant production of amylase and polygalacturonase by *T. viride* BITRS-1001 in submerged culture.

Of the nitrogen sources tested, the organic nitrogen substrates, peptone and casein, supported better biomass yield and enzyme activity of the fungus as compared to the inorganic nitrogen substrates tested. The observation is in agreement with [29] which reported that organic nitrogen sources supported the good growth of fungi more than inorganic nitrogen sources. Vahidi et al. [43] reported that good growth and antifungal activities were observed when complex nitrogen sources—yeast extract, peptone—were used compared to inorganic nitrogen source (NH_4_Cl and NaN0_3_). Akhilesh et al. [9] equally reported best polygalacturonase production with *Mucor circinelloides* ITCC 6025 when casein hydrolysate and yeast extract were used together, while Sasi et al. [41] reported that organic nitrogen induced the highest amylase activity in estuarine strain of *Aspergillus* spp. This preponderance of organic nitrogen sources on inorganic sources might be due to the fact that the organic nitrogen sources were better good growth stimulators. During growth and enzyme production, the fungus strain probably hydrolyzed the organic nitrogen releasing their mineral component and other growth factors in them into constituents that can be easily incorporated into cellular metabolism [31].

Factors like carbon and nitrogen sources and their concentrations have always been of great interest to the researchers in the enzyme industry for the low-cost media design. It is also known that 30–40% of the production cost of industrial enzymes is estimated to be the cost of growth medium. Therefore, it is of great significance to optimize the conditions for cost-efficient enzyme production [26]. However, investigations on the impact of carbon and nitrogen supplements had revealed that not all carbon and nitrogen sources would act as enhancer for simultaneous production of these enzymes in a single fermentation system. Unlike in single-enzyme production, role of supplements becomes very critical in multienzyme production as not many supplements enhance simultaneous production of all enzymes in a single bioreactor [27]. The present study thus indicated that *T. viride* BITRS-1001 produced high amounts of amylase and polygalacturonase in minimal medium, which has been modified with certain carbon and nitrogen sources concomitantly. So, it is concluded that minimal medium can be used under submerged fermentation for the concomitant production of amylase and polygalacturonase in submerged cultivation. Production of amylase and polygalacturonase in single fermentation can be particularly effective and useful for industries where both these enzymes are used together, such as food, animal feed, and textile. Further experiments will, however, have to be done to purify the secreted amylase and polygalacturonase as well as stability studies will have to be performed to enhance the application of enzyme to commercial level.
